# SubRecon: ancestral reconstruction of amino acid substitutions along a branch in a phylogeny

**DOI:** 10.1093/bioinformatics/bty101

**Published:** 2018-02-28

**Authors:** Christopher Monit, Richard A Goldstein

**Affiliations:** Division of Infection and Immunity, University College London, London, UK

## Abstract

**Summary:**

Existing ancestral sequence reconstruction techniques are ill-suited to investigating substitutions on a single branch of interest. We present SubRecon, an implementation of a hybrid technique integrating joint and marginal reconstruction for protein sequence data. SubRecon calculates the joint probability of states at adjacent internal nodes in a phylogeny, i.e. how the state has changed along a branch. This does not condition on states at other internal nodes and includes site rate variation. Simulation experiments show the technique to be accurate and powerful. SubRecon has a user-friendly command line interface and produces concise output that is intuitive yet suitable for subsequent parsing in an automated pipeline.

**Availability and implementation:**

SubRecon is platform independent, requiring Java v1.8 or above. Source code, installation instructions and an example dataset are freely available under the Apache 2.0 license at https://github.com/chrismonit/SubRecon.

## 1 Introduction

An evolutionary biologist may notice that taxa within a single clade in their sequence dataset possess a distinctive characteristic, such as a unique function. They may wish to investigate the evolutionary events occurring on the ancestral branch dividing this clade from other nodes in the phylogeny, by determining how the ancestral states changed between the two nodes on either side of that branch.

Two ancestral reconstruction techniques are widely used and address distinct statistical questions. *Joint* reconstruction estimates the set of character states for all internal nodes, reconstructing the whole history of states in the phylogeny (Pupko *et al.*, 2000; Yang *et al.*, 1995). *Marginal* reconstruction estimates states at a single internal node of interest, without conditioning on states at other internal nodes (Koshi and Goldstein, 1996; Yang *et al.*, 1995). Marginal reconstructions of states at two adjacent nodes will not provide a valid indication of the changes that occurred along the branch connecting them, as the independently estimated states may be incompatible. A complete joint reconstruction provides estimates conditional on the states of all other nodes of the tree, biasing the reconstruction at the nodes of interest.

We have developed a hybrid technique that overcomes these limitations by jointly reconstructing states at nodes either side of a single branch, while marginalizing over states at other internal nodes. We present a convenient implementation, SubRecon, which performs this reconstruction for amino acid states. SubRecon is simple to both install and run, has intuitive, configurable output and is suitable for large datasets.

## 2 Materials and methods

### 2.1 Theory

We model sequence evolution as a site-independent, time-continuous, reversible Markov process (see, e.g. Yang, 2014). Our approach is applicable to nucleotide, codon or amino acid states, but our implementation considers the latter only. For a given alignment site, we calculate the joint probability of a pair of states at the internal nodes either side of a branch of interest, while marginalizing over states at other internal nodes in the phylogeny. This is conditional on states observed at the tip nodes (data, *D*), a known or estimated phylogeny topology and a substitution rate matrix ***Q***, with state equilibrium frequencies π defined empirically or estimated previously.

Let *A* and *B* be the internal nodes connected by the branch, which is of length *t*, and let *a* and *b* represent possible states at these nodes. We use $P{\left(t\right)}_{ab}$ for the *a* to *b* transition probability along the branch, where P(t)=exp ⁡(Qt). Let $\pi_{x}$ be the equilibrium probability of state *x* and $P(D_{X}|x,\theta )$ the probability of the data *D_X_* for nodes descending from node *X*, conditional on model parameters *θ* and state *x* at *X*, while marginalizing over states at other internal descendant nodes, computed with the pruning algorithm (Felsenstein, 1981). We position the root node on the branch of interest, meaning the sets of taxa descending from *A* and *B* are mutually exclusive and together comprise all taxa in the phylogeny. The joint probability that states *a* and *b* existed at *A* and *B* respectively, marginalizing over other nodes, is thus

(1) $$\begin{array}{cc} & P(A=a,B=b|D,\theta )\\ & =\frac{\pi_{a}P{\left(t\right)}_{ab}P(D_{A}|a,\theta )P(D_{B}|b,\theta )}{\sum_{a\prime ,b\prime }\pi_{a\prime }P{\left(t\right)}_{a\prime b\prime }P(D_{A}|a\prime ,\theta )P(D_{B}|b\prime ,\theta )}. \end{array}$$

The root position and designations of *A* and *B* and are arbitrary since the process is reversible: $\pi_{a}P{\left(t\right)}_{ab}=\pi_{b}P{\left(t\right)}_{ba}$. The denominator is equal to the marginal probability of the data given the model; i.e. the likelihood, P(D|θ). The *a* and *b* pair maximizing P(A=a,B=b|D,θ) is preferred.

Equation 1 assumes a single substitution rate for each site analyzed, but this is unrealistic (see Yang, 2014). We therefore extend Eq. 1 to include the discrete approximation for gamma-distributed rates, as is commonly used in phylogenetic analysis implementations (e.g. Stamatakis, 2014; Yang, 2007). We allow *k* classes, each with rate *r_i_*, i∈1,2,…,k. We assume the gamma distribution shape parameter *α* has been estimated (the scale parameter *β* is set equal to *α*, by convention). Let $P(D_{X}|x,\theta ,r_{i})$ be defined as above, but where the length of each branch descending from node *X* is multiplied by *r_i_*. Then,

(2) $$P(A=a,B=b|D,\theta ,\alpha )=\frac{\sum_{i}^{k}\pi_{a}P{\left(tr_{i}\right)}_{ab}P(D_{A}|a,\theta ,r_{i})P(D_{B}|b,\theta ,r_{i})}{\sum_{i\prime ,a\prime ,b\prime }\pi_{a\prime }P{\left(tr_{i\prime }\right)}_{a\prime b\prime }P(D_{A}|a\prime ,\theta ,r_{i\prime })P(D_{B}|b\prime ,\theta ,r_{i\prime })}.$$

### 2.2 Simulations

**Fig. 1.**
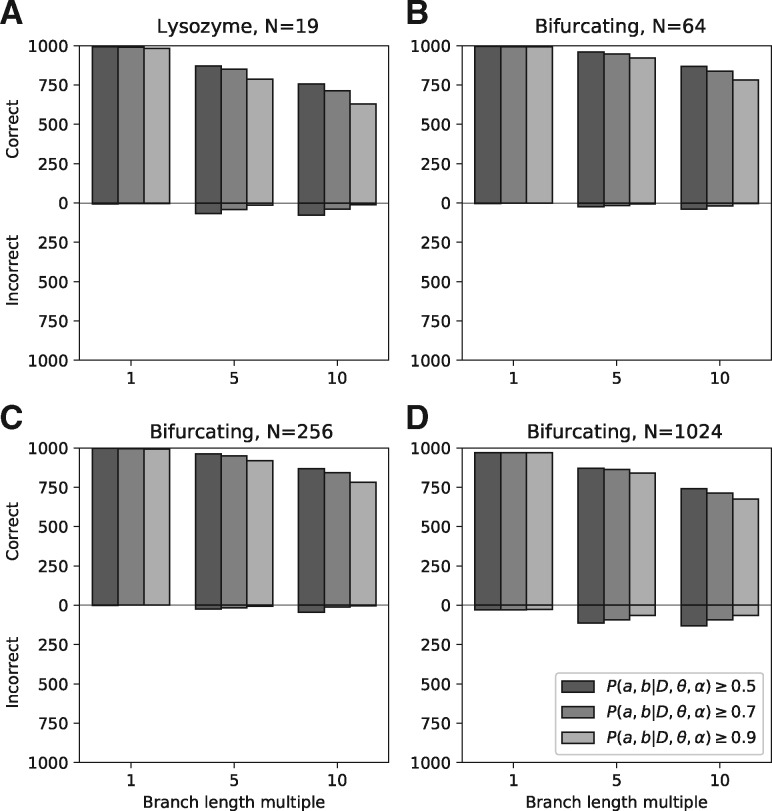
Accuracy of SubRecon over a range of dataset sizes and branch lengths. We first estimated a phylogeny, π and *α* for 19 primate lysozyme protein sequences (Messier and Stewart 1997), using RAxML and WAG substitution model with 4 gamma-distributed rate categories. We then simulated evolution of 1000 sites using WAG and these parameters using Evolver (Yang, 2007), with input branch lengths multiplied by 1, 5 or 10. (**A**) The number of sites where SubRecon’s max⁡[P(A=a,B=b|D,θ,α)] estimated both *a* and *b* correctly (upper bars) or incorrectly (lower bars) using a range of minimum probability thresholds, for the branch ancestral to the Colobines (*N* = 5). (**B–D**) Further simulations used arbitrary bifurcating topologies containing 64, 256 or 1024 taxa with equal branch lengths, chosen such that their sum per taxon was equal to that of primate lysozyme (0.392/19≈0.02) and then multiplied by 1, 5 or 10. The chosen branch of interest was that ancestral to 25% of taxa

Simulation experiments using various phylogeny topologies, branch lengths and minimum probability thresholds show reconstruction estimates to be accurate and powerful. For mid-sized datasets (Fig. 1A–C) the max⁡[P(A=a,B=b|D,θ,α)]≥0.9 threshold yielded between 0 and at most 15 inaccurate reconstructions out of 1000, while even the 0.7 threshold provided a reasonable tradeoff. For very large, highly divergent datasets where the branch of interest is distant from terminal taxa (as in Fig. 1D), high minimum thresholds are advisable.

### 2.3 Software implementation

SubRecon computes P(A=a,B=b|D,θ,α) for all *a* and *b* pairs, for a specified pair of adjacent internal nodes, using any of several amino acid empirical substitution models; e.g. WAG (Whelan and Goldman, 2001), implemented in PAL (Drummond and Strimmer, 2001). The phylogeny, including branch lengths, and gamma distribution shape parameter (*α*) should be estimated in advance using popular phylogeny estimation tools, such as RAxML (Stamatakis, 2014). The models’ default equilibrium frequencies (π) can be used or estimated values provided. SubRecon is designed to handle large datasets, as multiple sites can be analyzed in parallel with a user-defined number of computing threads, while log-transformations prevent numerical underflow errors.

Written in Java v1.8, SubRecon is platform independent and we include build scripts allowing easy compilation using Apache Ant (http://ant.apache.org). Its command line interface (based on jCommander, http://jcommander.org) is simple and the output is intuitive yet amenable to parsing by downstream software in an analysis pipeline. The detail and formatting of output can be controlled by the user.

## 3 Conclusion

Existing reconstruction implementations are not well suited to comparing ancestral states underlying phylogenetically and biologically distinct taxa in a protein sequence dataset. Our technique combines joint and marginal reconstruction approaches, allowing efficient and valid comparisons. Our convenient implementation, SubRecon, should be a useful addition to the toolkit of the investigator studying comparative evolutionary biology.

## Funding

This work was supported by the UK Medical Research Council and the UK Biotechnology and Biological Sciences Research Council [grant numbers MC_U117573805, BB/P007562/1].

*Conflict of Interest*: none declared.
